# LifeMap Discovery™: The Embryonic Development, Stem Cells, and Regenerative Medicine Research Portal

**DOI:** 10.1371/journal.pone.0066629

**Published:** 2013-07-17

**Authors:** Ron Edgar, Yaron Mazor, Ariel Rinon, Jacob Blumenthal, Yaron Golan, Ella Buzhor, Idit Livnat, Shani Ben-Ari, Iris Lieder, Alina Shitrit, Yaron Gilboa, Ahmi Ben-Yehudah, Osnat Edri, Netta Shraga, Yoel Bogoch, Lucy Leshansky, Shlomi Aharoni, Michael D. West, David Warshawsky, Ronit Shtrichman

**Affiliations:** 1 LifeMap Sciences LTD, Tel Aviv, Israel; 2 LifeMap Sciences Inc, Alameda, California, United States of America; 3 BioTime Inc. Alameda, California, United States of America; Instituto Butantan, Brazil

## Abstract

LifeMap Discovery™ provides investigators with an integrated database of embryonic development, stem cell biology and regenerative medicine. The hand-curated reconstruction of cell ontology with stem cell biology; including molecular, cellular, anatomical and disease-related information, provides efficient and easy-to-use, searchable research tools. The database collates *in vivo* and *in vitro* gene expression and guides translation from *in vitro* data to the clinical utility, and thus can be utilized as a powerful tool for research and discovery in stem cell biology, developmental biology, disease mechanisms and therapeutic discovery. LifeMap Discovery is freely available to academic nonprofit institutions at http://discovery.lifemapsc.com

## Introduction

Understanding how pluripotent stem and downstream progenitor cells differentiate into mature functional cells during embryonic development is of fundamental interest, with broad applications in fields as diverse as regenerative medicine, teratology and cancer [1]. The ability to generate, or regenerate functional cells and tissues from stem and progenitor cells by manipulating them *in vivo* or controlling the *in vitro* differentiation, is one of the greatest promises for medicine in the coming decades, providing a broad array of applications that have the potential to transform the field of medicine.

Several strategies are being developed to achieve better control over the process of generating the desired target cells and tissues. These strategies utilize e.g. plasticity [2] – the ability of a cell to change its fate in response to extra-cellular signals and niche effects to induce resident tissue-specific adult stem cells (*in vivo* and *in vitro)*. More explicit strategies include *in vitro* differentiation of embryonic stem (ES) cells, and reprogramming of cells to produce induced pluripotent stem (iPS) cells, collectively referred to herein as pluripotent stem (PS) cells. Clearly, the knowledge that is essential in these strategies and methods spans traditionally distinct disciplines – developmental biology, stem cells biology and general cellular and molecular biology.

The numerous stem cell types and second generation embryonic progenitor cells differentiation protocols, as well as innovative ways of managing the processes of differentiation, isolation and propagation of novel cells, and *in vivo* lineage tracing reports suggest that the field of regenerative medicine will continue to be challenged with the enormous complexity of unique cell types existing distinctly in the developing organism.

During the differentiation process of human PS cells leading to the hundreds of known derivatives, the cells transition and passage through intermediate progenitor cell phenotypes such as paraxial mesoderm, somatic mesoderm, migrating neural crest, and so on. However, very little is known about the molecular markers, cell culture requirements, specific protocols for differentiation, or replicative capacity of most of these intermediate embryonic progenitor cell types. In addition, first-generation hES cell-based therapeutic candidates are likely contaminated with various embryonic progenitors, the capacity of such contaminants to generate various types of derived cells and possible adverse effects is currently a matter of considerable debate.

*In vitro* stem cell differentiation and *in vivo* differentiation during development are closely related, yet that relation is not trivial or simple to mimic. Nonetheless, formation of tissues and organs that occurs *in vivo*, such as in transgenic murine models of development may provide highly valuable clues about developmental paths, signaling and gene expression patterns. To provide the wide scope and essential level of detailed information needed for improving stem cell applied and basic research, LifeMap Discovery integrates embryonic development and stem cell biology with molecular, cellular, anatomical and disease-related information into an interlaced data model that allows integration of these distinct areas. The underlying postulate made by LifeMap Discovery, is that the gene expression patterns that occur in developing cells, and the signaling between cells that regulates cell differentiation, provides invaluable information for (i) biologically accurate identification and classification of differentiated stem and progenitor subtypes, and (ii) suggesting mechanisms and enable the researcher to deduce the best protocols for differentiating these cells to the desired target phenotype.

In the stem cell field, very few publicly available databases currently exist; each of the existing databases targets certain elements of interest to stem cell research such as expression, regulation [3], [4] [5] or attempted computational classification [4]. However, currently, no resource provides a comprehensive set of information and supporting data needed to study and develop stem cell applications. In the area of developmental biology, databases have been available for a number of years. Mouse is the most widely studied animal model and the e-Mouse Atlas Project (EMAP) [6] and Mouse Genome Informatics (MGI) [7] databases are two central resources, both using a similar hierarchical ontology describing the developing mouse originally developed by the EMAP. These databases provide essential information about the gross mouse anatomy and detailed histological structure, and a framework into which information about gene function can be mapped. The EMAP ontology is extremely useful and widely used; it supports certain aspects of the developmental-centric cellular level ontology that is required to describe cellular differentiation, and composed of snapshots at particular mouse development stages with links between anatomical structures at different developmental times. In addition to the above comprehensive resources, a number of databases exist specializing in organism [8], organ [9], [10] or developmental time [3].

LifeMap Discovery is designed to provide the research community with viable, scalable, and easy to manage data describing cellular and anatomical development, and presented in a useful framework model, and with various types of *in vitro* cells providing molecular and cellular information such as gene expression, culturing conditions, differentiation protocols and related cell-therapy applications about these entities. Additionally, the developmental *in vivo* data and the experimental *in vitro* data are manually inter-linked according to their relevance to provide users with the full array of relevant information for optimal stem cell and developmental biology-related research.

## Results and Discussion

### Database structure

**Figure 1:**
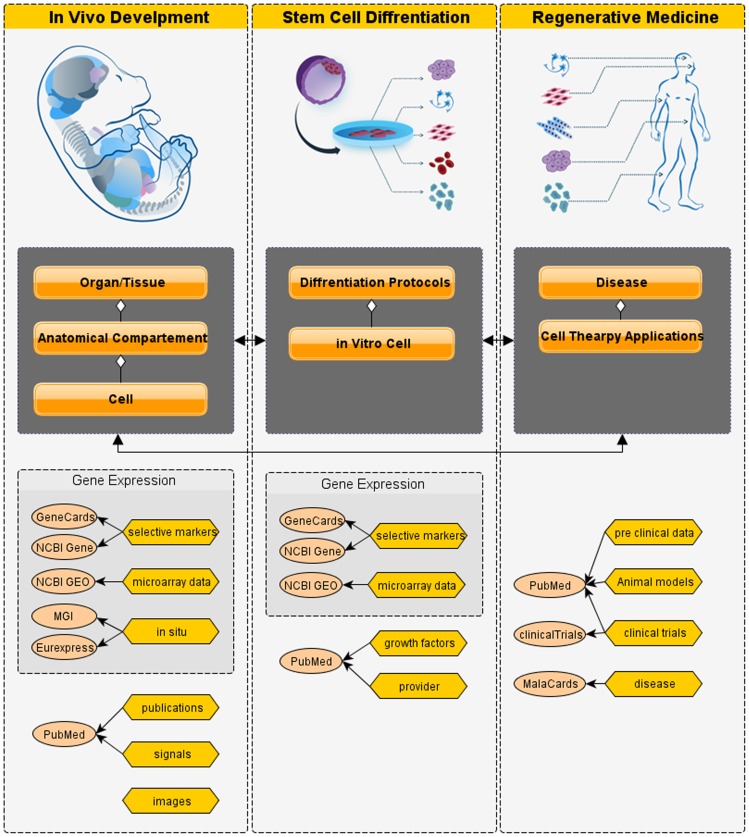
Database Structure. (A) The Discovery database contains three main components, (i) In vivo development, (ii) Stem cell Differentiation, and (iii) Regenerative Medicine. (B) Sections within each main component (e.g. the In Vivo Development component is made up of cells contained in anatomical compartments that in turn are contained in organs/tissues. The sections are connected by reciprocal links (black arrows). (C) Within each database component, main data categories and their main source of references are listed, e.g. inside Stem Cell Differentiation, growth factors are listed and linked to PubMed citations.

LifeMap Discovery is based on systematic gathering, analysis and *de novo* assimilation of peer reviewed scientific data and data resources describing mouse and human development. Figure 1 illustrates the overall database structure and their interrelations. The database is constructed from the following components:

1. *In vivo development* – cellular and anatomical ontology of the mammalian body.
2. Stem cell differentiation – describing cultured cells and differentiation protocols.
3. Regenerative medicine – utilizing stem cells for development of therapeutic products.

These different parts are evaluated and inter-linked by computational and manually curated methods, most noteworthy, the *in vivo* cells and anatomical compartments are linked to their most similar *in vitro* entities, based on gene expression analysis or manual assignments based on literature.

### *In vivo* development

**Figure 2:**
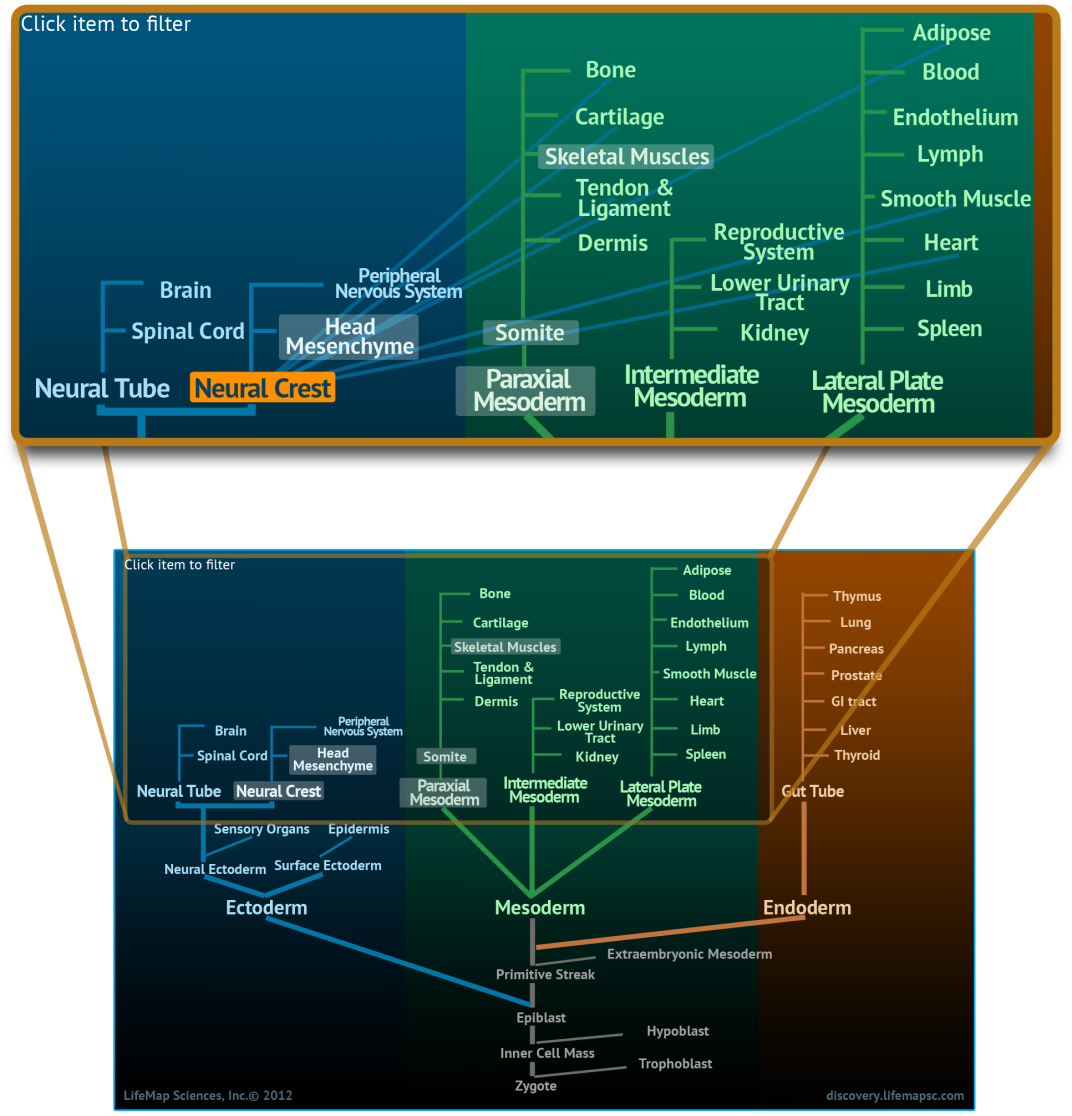
The Developmental Ontology Tree. An interactive viewer available at the organ/tissue level. The ontology tree presents a low-resolution overview of organ/tissue development, and facilitates navigation by clickable entities that open the related card. The lower image shows the full ontology tree starting from the zygote, through the three germ layers; ectoderm, mesoderm and endoderm and to their derived organs and tissues. The upper image is an enlargement showing the contribution of the neural crest to the development of multiple tissues such as bone, cartilage and skeletal muscle as it is displayed in the database.

Underlying LifeMap Discovery is the ontology of the cellular differentiation that occurs during mammalian embryonic development. Decades of efforts in experimental embryology have elucidated the majority of the cellular pathways of mammalian development; hence, substantial amounts of data can currently be summarized in a relational database connecting each developing cell to a specific, temporospatial anatomical compartment that composes the developing organ or tissue. To account for these complex relations, and how data is collected and recorded in scientific literature, embryonic development is presented in LifeMap Discovery on three concentric levels:

1. **Organ/Tissue:** A low-resolution description of mammalian development, beginning at the zygote, proceeding to the three germ layers as well as extraembryonic tissues, and extending towards the derived organs and tissues that comprise the adult body (Figure 2). A tissue is defined as a collection of cells that perform specific functions, and may be found in multiple body regions, e.g. cartilage, bone, skeletal muscle etc. An organ is defined as a collection of tissues and specialized cells, joined in a structural unit to serve common functions, e.g., kidney, heart, liver.
2. **Anatomical Compartment:** The developmental ancestry of specific temporospatial regions within an organ/tissue. At this level, the entities are connected to the related organ/tissue and can be linked to one or more parent compartments (which may be located outside of the organ/tissue). Anatomical compartments are constructed as a developmental tree, where each parent compartment gives rise to one or more compartments, i.e. the child has a “developed-from” relation to its parent. In addition, some anatomical compartments are sub-divided, or contain other anatomical compartments, i.e., a “part-of” entity relation.
3. **Cell:** Cellular data is collected at specific developmental time points or embryonic stages (e.g. Theiler stage for mouse or Carnegie stages for human). Detailed developmental paths taken by cells directed toward specific fates, such as blood, endothelium, skeletal muscles, bone, cartilage etc. Somatic cells transverse a developmental path, which defines their cellular ontology from their first ancestor or ancestors (termed “primary progenitor”) to the fully differentiated cell type.

**Figure 3:**
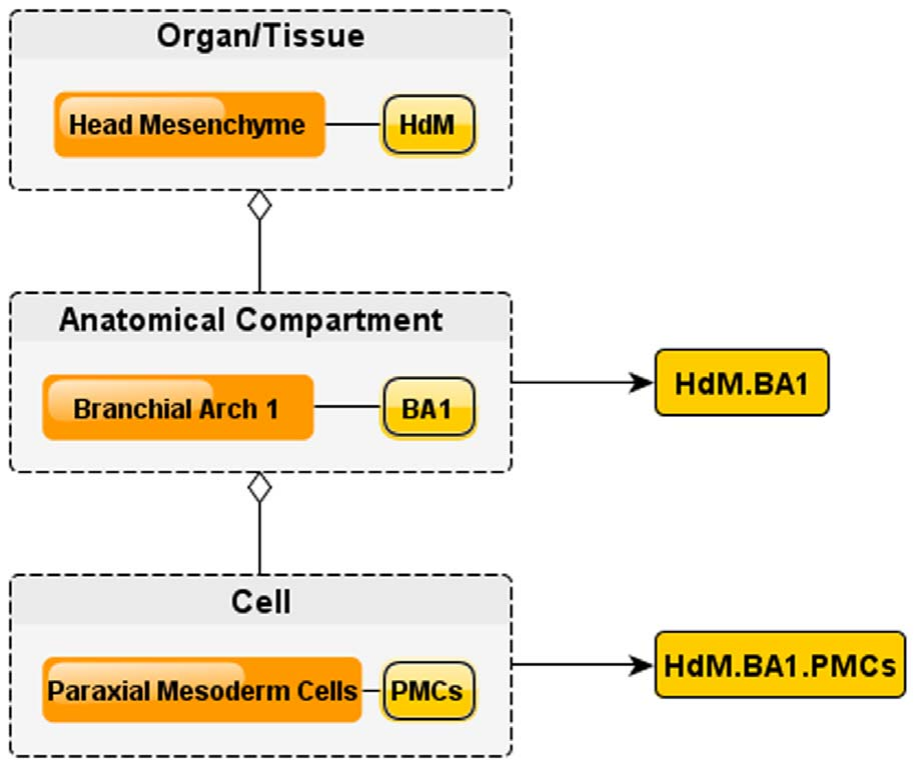
Assignment of Embryonic Index (EIndex). Entities in each embryonic level are assigned a local index, e.g. the Head Mesenchyme local index is HdM, and one of its anatomical compartments, Branchial Arch 1 local index is BA1. The full Embryonic index for Branchial Arch 1 is hence HdM.BA1. Similarly, Paraxial Mesoderm Cells contained in the Branchial Arch 1 are given a local index PMCs and a fully qualified EIndex of HdM.BA1.PMCs.

Capturing the complete cell differentiation process into an ontology also requires the ability to address each of the cells and anatomical structures in a non-redundant fashion. LifeMap Discovery introduces a cell nomenclature system that takes advantage of this three-tier system and distinctly identifies the cell-anatomical relation in the developing embryo so that ambiguities are effectively eliminated. Each cell obtains a unique ID referred to as the Embryonic Index (EIndex). The EIndex is generated by combining indexes from the organ/tissue, anatomical compartment and cells. Figure 3 illustrates construction of the full EIndex of paraxial mesoderm cells (local index: PMCs) which populate the branchial arch 1 anatomical compartment (EIndex: BA1), related to the head mesenchyme organ (EIndex: HdM) is: HdM.BA1.PMCs. Note that a cell can be related to several developmental paths, but it has a unique anatomical localization described by the EIndex. e.g: the mentioned cell: HdM.BA1.PMCs [11] is the first ancestor (the primary progenitor) of both skeletal muscle and related bone lineages of the developing skull. Each of the above entities (organ/tissue, anatomical compartment and cell) has a corresponding web page or “card” in LifeMap Discovery. The card provides a general description of its developmental function and structure.

The organ/tissue card contains an illustration of the organ and links to related organs, unique lineages (e.g.: the motor neurons lineage is available under the spinal cord organ card), anatomical compartments and cells, differentiation protocols and cell therapy applications.

The anatomical compartment card provides a brief description accompanied by illustrations describing development, structure and anatomy as well as links to cells that populate the specific anatomical compartment. In addition, the card provides lists of genes specifically expressed in the compartment of interest and its related cells, links to data which has been extracted from filtered *in situ* hybridizations, and relevant high throughput experiments. The card also lists diseases and cell therapy applications related to the specific anatomical compartments or to its populating cells.

The cell card displays the relevant embryonic developmental stage, and provides a list of expressed genes which were gathered by manual curation and/or extracted from related microarray and/or *in situ* hybridization experiments. The cell card also provides information on signaling and signal transduction cascades which affect cell function and differentiation *in vivo*, as well as related diseases which affect or involve the given cell and can potentially lead to cell-based therapeutic application by related *in vitro* cells. A number of useful databases that host *in vivo* gene or protein expression data such as MGI [7], Eurexpress [3], and Human Protein Atlas [12] are linked from the cells and tissues to show data resulting from High-throughput gene expression data.

Figure 2 illustrates LifeMap Discovery's modeling of *in vivo* development, e.g. skeletal muscle development from paraxial mesoderm. In general, skeletal muscle development differs by the anatomical origin, i.e head or trunk (body) areas of the developing embryo. The described complexity is demonstrated by the highlighted entities in the high level-ontology tree, shown at Figure 2; at early stages of embryogenesis, the mesoderm formed during gastrulation is further developed into the paraxial mesoderm (along with the lateral and intermediate mesoderm) which later differentiates into somites ([13]) and to the un-segmented paraxial mesoderm of the head ([14]). The developing somites are epithelial structures which eventually give rise to many lineages such as cartilage, bone and most of the embryonic skeletal muscles. In contrast, in the head region, paraxial mesoderm resides alongside the cranial neural crest (NC) to collectively become 'head mesenchyme' which will give rise to the aforementioned tissues of the head ([15], [16]). NC is often referred to as the 4^th^ germ layer of the embryo, emerging early in mammalian development and dividing into head (cranial) and trunk populations. While the specialized paraxial mesoderm within this mesenchyme contribute to the development of head skeletal muscles (eye, jaw and neck), the NC will differentiate into multiple cell types including bone, cartilage, and peripheral nerves of the anterior aspects of the head ([15],[17],[18],[19],[16]) (Figure 2, Upper panel).

**Figure 4:**
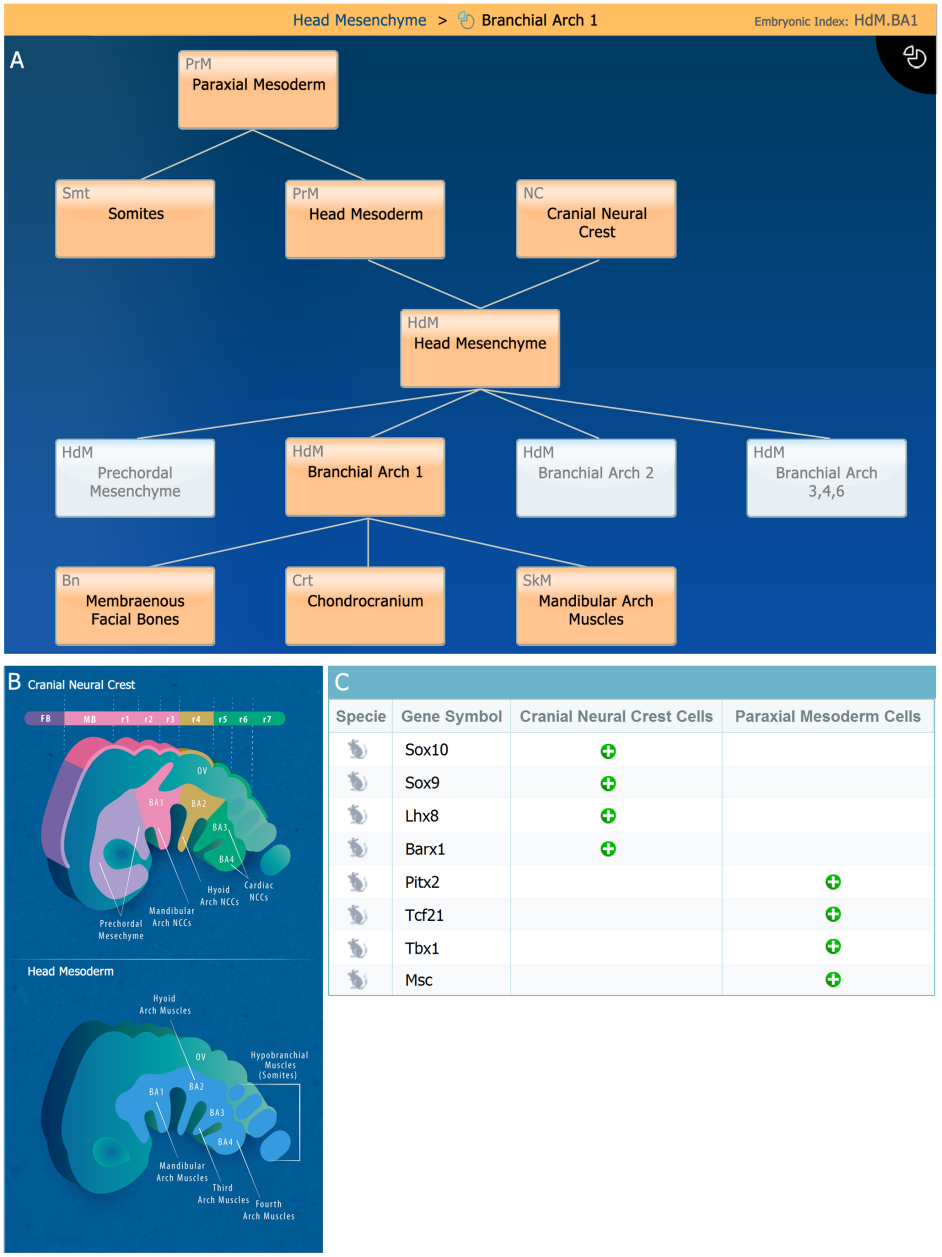
Branchial Arch 1 (HdM.BA1) anatomical compartment development card. **A.** Anatomical compartment viewer demonstrating the contribution of ‘Head Mesoderm’ and ‘Cranial Neural Crest’ to the developing ‘Head Mesenchyme’, which give rise to Branchial Arch 1 and its functional derivatives (i.e., cartilage, bone and skeletal muscles), is depicted in this viewer (highlighted, orange colored boxes). **B.** Illustration showing the embryonic ontology and development of the two main cellular components (i.e., paraxial mesoderm and neural crest cells) which give rise to skeletal elements of the head (lateral view). Such anatomical illustrations are available in the database at the organ/tissue and anatomical compartment cards **C.** Examples of the display of selective gene markers of cells populating the anatomical compartment. Here the selective genes of CNC cells and Paraxial Mesoderm cells are shown in the BA1 anatomical compartment card. **Abbreviations**: mb, midbrain; fb, forebrain;r1–7, rhombomeres 1–7; HdM.BA1–4, branchial arch 1–4; OV, optic vesicle.

Somites and head mesenchyme are useful examples demonstrating the anatomical compartment level. During embryonic development, the epithelial somite gives rise to two anatomical compartments termed dermomyotome and sclerotome (for examples of these compartments see: [20] and [21], respectively). The dermomyotome will further differentiate and contribute to the formation of skeletal muscles and the sclerotome will give rise to axial bones and cartilage elements [22]. Within the head region, neural crest and mesoderm (i.e., ‘head mesenchyme’) will form the branchial arch 1 (BA1) anatomical compartment (HdM.BA1; Figure 4A; also depicted in Figure 4B). HdM.BA1 is a transient structure that will form the template of the jaws in terms of skeleton and its associated musculature. Concomitantly, the contribution of both neural crest and mesoderm to HdM.BA1 molecular gene expression is demonstrated in Figure 4C.

**Figure 5:**
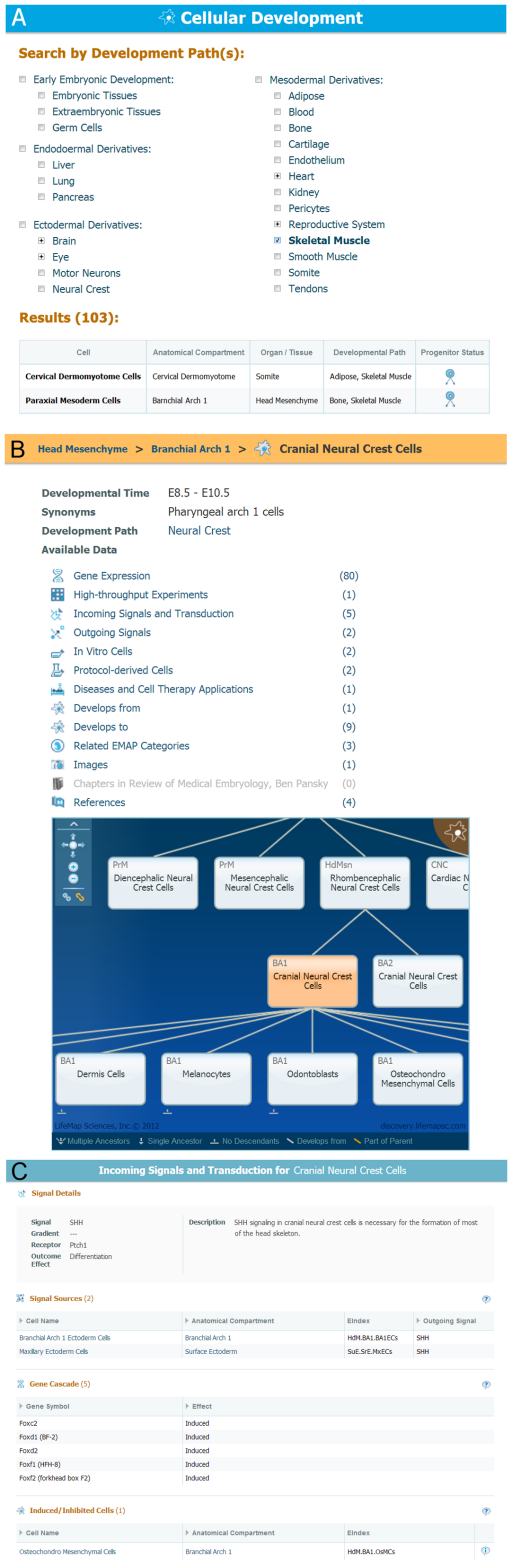
Cellular Development level and Cell Card Representation. This figure demonstrates some of the comprehensive data provided for a single cell in the database. **A.** The cellular filter list, available at LifeMap Discovery, showing all available cellular developmental paths currently available in the database. This example demonstrates results from search of skeletal muscle-related cells; two selected cells with their developmental path annotation (e.g., Skeletal Muscle) are shown for simplification. **B.** ‘Cranial Neural Crest Cell’ (CNCCs) card shows the available information (e.g., gene expression) for these cells, accompanied by the interactive clickable graphical development viewer on the right. **C.** An example for a specific signal display- with a description (SHH), signal source, associated gene cascade and biological cellular outcome.

On a cell ontology level, LifeMap Discovery provides a browsing tool (Figure 5A) that lists all available developmental paths and allows various filtering. Following selection, the results panel presents all related cells sorted by order of their development, starting with the primary progenitors. As illustrated in Figure 5A, a selection of skeletal muscle results with a summary table containing one hundred skeletal muscle related cells; here, two unique muscle progenitors appear: cervical dermomyotome cells of the somite and Paraxial mesoderm cells of the Head mesenchyme. Both of these cells are the primary progenitors of skeletal muscle in the head (‘head mesenchyme’) and in the trunk (‘somite’) lineages respectively.

Head paraxial mesoderm cells ([11]), which eventually give rise to head musculature, express unique regulators such as *Pitx2, Capsulin (Tcf21), Tbx1, MyoR (Msc)* as shown in Figure 4C, depicting the HdM.BA1 anatomical compartment card. These selective markers are specific for the head progenitors and are not expressed by the trunk progenitors. Moreover, the head progenitor cells lack expression of the early muscle master regulator *Pax3*, which was shown to be essential in trunk myogenesis (together with the muscle regulatory factor Myf5). Specific differentiation signals such as Wnt3A and Wnt1, specifically inhibit skeletal muscle progenitors' differentiation in the head region ([23]), while prompting their differentiation in the trunk ([20]). Thus, full multi-dimensional perspective of the cell development path is adequately displayed in LifeMap Discovery.

Another example of the cellular resolution available in LifeMap Discovery is demonstrated by the cranial neural crest (CNC) cells [24], which eventually give rise to most of the head skeletal elements (e.g., skull bones). These cells will specify to become osteochondral mesenchymal cells during early mouse development (Figure 5B; [25]). Among other signaling cascades, Sonic Hedgehog (SHH) induces differentiation of Cranial Neural Crest Cells (CNCCs) into the specific skeletal fates (Figure 5C). Along with early neural crest selective markers (i.e., a ‘selective marker’ annotation indicates genes that have been suggested to be characteristic to the cell) are; *Sox10*, *Twist1*, *Dlx5* and *Sox9,* which was shown to be also expressed in other embryonic tissues. Site specific homeobox genes, such as *Lhx8 and Barx1,* are specifically expressed in CNCCs within BA1 (Figure 4C). Finally, these progenitors will differentiate into functional cartilage and bone cells which are specific to the head region (e.g., intramembranous bones). In addition, available and related *in vitro* cells are provided in this cell card, CNC differentiation protocols and annotated disease (Figure 5C).

The database comprehends the biological complexity utilizing the three-level tier system in an attempt to accelerate researchers search for information. Here, the two different cells described in the examples (paraxial mesoderm cells and CNC cells) are located at the same anatomical compartment, HdM.BA1, yet contribute to different cellular developmental paths, with distinct progeny and unique molecular signatures.

### Stem cell differentiation

LifeMap Discovery provides information for various types of *in vitro* cells including embryonic stem cells (ES cells), induced pluripotent stem cells (iPSCs), embryonic progenitor cells, fetal and adult stem cells and primary cells. These cells are designated in the database as either (i) source cells, undergoing any number of differentiation protocols or cell therapy applications, or (ii) protocol derived cells (PDCs) that are generated by a single differentiation step, usually as part of a multistep differentiation protocol.

The “stem cell” differentiation section includes two main levels of information discussed in detail below:

1. Stem, progenitor and primary cells [26]
2. Differentiation protocols [27]

### Stem, progenitor and primary cells

This section provides information relating to the various *in vitro* cells which may serve as source cells for differentiation protocols and cell therapy applications or as valuable *in vitro* cell data source containing high throughput data that is useful for classification purposes. Similar to *in vivo* cells, each *In vitro* cell is presented in a card including information about its growth conditions, gene expression, related cell-therapy applications, references and more. Source i*n vitro* cells may be matched to one or more *in vivo* cells or anatomical compartments of the embryo or mature mammal. The matching is done either manually or computationally (see section below), and is primarily based on gene expression profiles, but can also be based on function and morphology observations described in the literature.

The main cell types available in this section are:

1. **ES cells and iPS cells:** Pluripotent cells from mouse or human origin, derived either from early stage embryos (ES cells) or from adult/fetal tissues by induction (iPS cells). Their ability to differentiate into mature cells derived from all three germ-layers makes them promising candidates to be used in regenerative medicine. Both cell types serve as source cells for differentiation protocols towards various mature cell types.
2. **Fetal/stem progenitor cells:** These cells are isolated from adult or fetal tissues and have limited differentiation capacity relative to ES cells or iPS cells. However, the ability to isolate them directly from patients make these cells attractive candidates for personalized regenerative medicine approaches. In LifeMap Discovery, these cells are described as source cells for both differentiation protocols and cell therapy applications.
3. **Primary cells:** These cells are isolated from adult tissues, have limited propagation capacity *in vitro* and do not possess differentiation potential. They are commonly used either as supportive cells for differentiation of stem cells, as source cells for cell replacement therapies or as a source of valuable data that may be useful in characterizing unknown cells. These cells are presented in LifeMap Discovery as sources of cell therapy applications. Their gene expression profiles are useful for identification of unknown differentiated cells.
4. **Embryonic progenitor cells:** Cells corresponding to the proliferating progenitors derived from pluripotent stem cells, are designated “embryonic progenitors in LifeMap Discovery. Clonally-purified embryonic progenitors are commercially available as PureStem™ human embryonic progenitor (hEP) cells, which were derived *in vitro* from hES cells, using a “shotgun” strategy including; spontaneous differentiation of hES cells into embryoid bodies (EBs), single cell dissociation and proliferation of various clones using a matrix of time periods and growth conditions [28]. This strategy yielded at least 140 distinct types of isolated clonal hEPs, genomically stable and lacking tumorigenicity, which makes them ideal candidates for regenerative medicine and stem cell research. High-throughput microarray analysis revealed that each of the hEPs express a unique set of gene markers, surface antigens and growth factors and therefore can be matched to specific cells in the *in vivo* developmental lineages ([29]). For instance, this strategy revealed that the 7PEND24 PureStem cells expresses the two site-specific mesenchymal gene markers LHX8 and BARX1 (previously shown to be selective markers of HdM.BA1), and shows evidence of regenerating articular cases *in vivo* [30]. A variety of PureStem progenitors are commercially available at http://bioreagents.lifemapsc.com.

**Figure 6:**
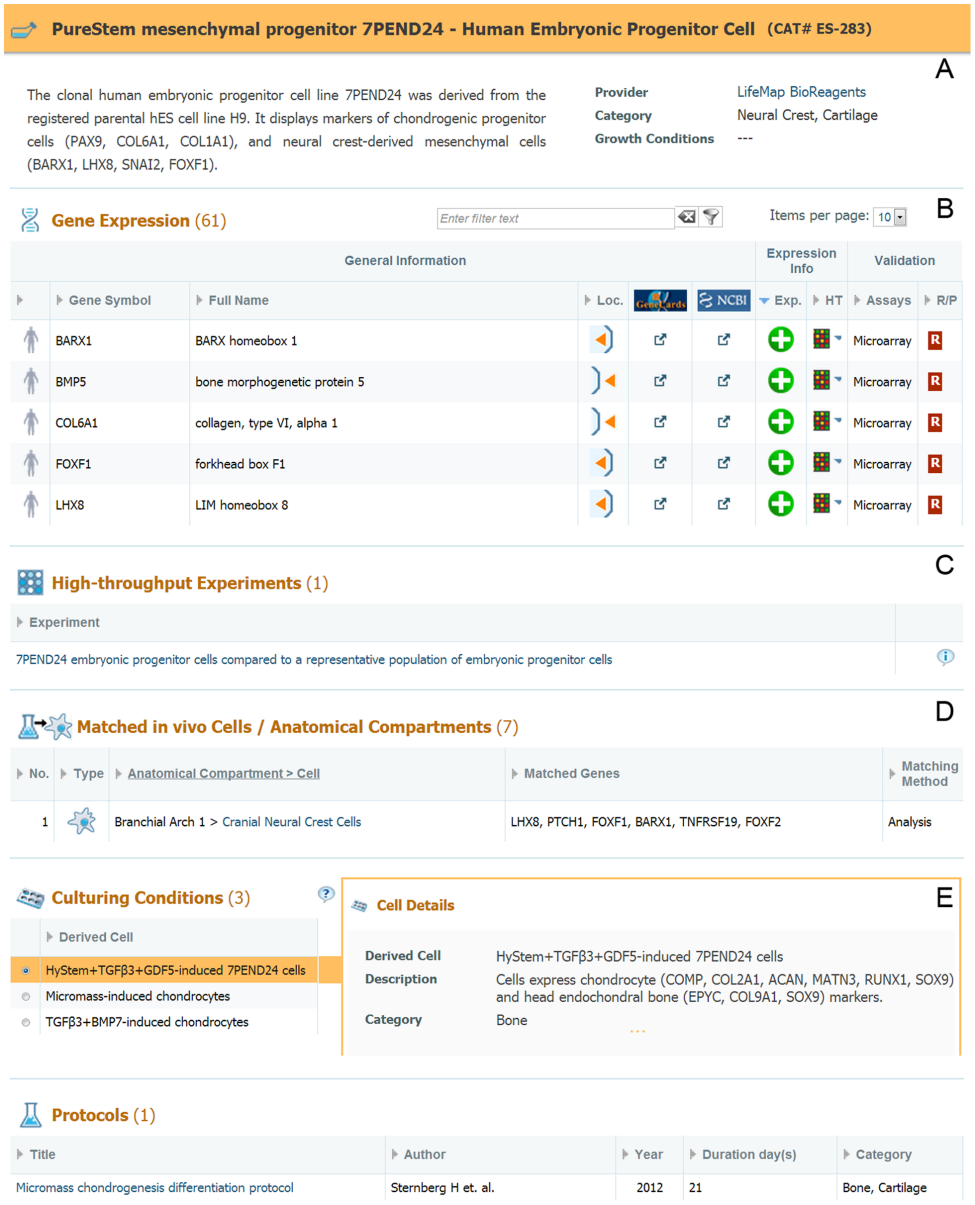
7PEND24 PureStem Progenitor Cell Card. **A**. Cell description and available data summary. **B.** Gene expression list including expression pattern, assay type and links to external resources. **C.** Links to cell-related high-throughput experiments, available in the database. **D.** List of *in vivo* cells or anatomical compartments that were matched to the PureStem progenitor and the related genes for each match**. E.** List of culturing conditions and protocols related to the PureStem progenitor cell.

The PureStem mesenchymal progenitor 7PEND24 card is shown in Figure 6, containing a short description of the cell's main characteristics and selective markers, followed by a short summary of the available data in the card (Figure 6A). The card includes a list of differentially expressed genes (Figure 6B) extracted from a microarray experiment where the cells were analyzed versus a general reference, as well as from manually curated selective markers (validated by qRT-PCR) such as: *LHX8, FOXF1*, *BARX1, FOXF2*, indicating the cell's similarity to neural crest cells. The card also provides a link to relevant high-throughput experiments presenting the gene expression profile of the progenitor line (Figure 6C). Gene expression -based computational matching to *in vivo* cells and anatomical compartments is shown at the matching section of the card (Figure 6D), indicating that the PureStem progenitor cells, 7PEND24, were matched to the above mentioned CNC cells (Eindex:HdM.BA1.CNCCs).

The card also shows different culturing conditions and protocols (Figure 6E) used to differentiate the PureStem progenitor cells, 7PEND24. For each treatment, the derived cell is described, as well as the growth conditions used for this derivation. The top 100 differentially expressed genes, including the selective markers for specific cell fates, are shown in the gene expression section. The related microarray experiment is available for each culturing method as well as matching to the closest comparable *in vivo* entity. Referring back to our example, treatment of the PureStem progenitor cells, 7PEND24, with HyStem (hyaluronan-based hydrogel) supplemented with TGFβ3, and GDF5 for 14 days, leads to differentiation into chondrocytes-like cells, based on the acquired expression of chondrocytes selective gene markers such as: ACVR1, RUNX1, PTH1R, FGFR3, SOX9, MATN3, COL11A2, LECT1, ACAN, COL9A2, COL2A1.

### Differentiation protocols

The stem cell differentiation protocols section provides a detailed flow of protocols used to induce differentiation of various pluripotent and multipotent stem and progenitor cells to clinically relevant target cells. Each protocol is displayed in a card that contains an informative and interactive viewer, outlining the protocol as a multistep process. The protocol card depicts the source and target cells of each step in the protocol and the details of the procedure required to obtain each target cell from its source cell. Each protocol is fully referenced (cited and linked to PubMed), and includes a concise description of each step. The main protocol section includes the “protocol derived cells”, in which a user can click on each intermediate cell type, and obtain all available information about the derived cell and the step details including description, duration, main growth factors, procedure, growth conditions and % efficiency. Genes expressed in the derived cell are shown, as well as the top differentially expressed genes, extracted from microarray experiments (when available) and linked to GEO [31]. The top ranking genes are based on the authors' paper, or calculated from normalized/raw data provided as supplementary data by the authors [32]. Based on the gene expression data, the source and target cells of the protocols can be matched to one or more *in vivo* cell entities in the database, as previously described.

**Figure 7:**
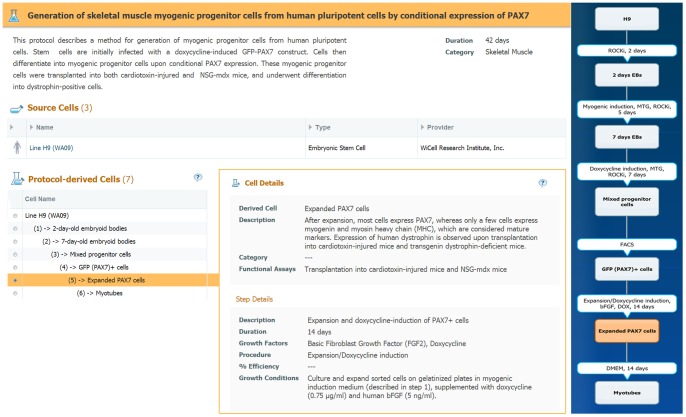
Differentiation Protocol Card. Synopsis of the protocol, including duration, a summary of available data (not shown in figure), and an interactive viewer (right hand side) showing the protocol flow and its differentiation steps. Information about the *in vitro* cells that are used as source cells for the protocol is presented below the protocol title. The list of the protocol derived cells (PDCs) are shown on the left, ordered by the protocol steps. For a selected PDC (highlighted in the diagram), a description for the cell and step details are available, including list of growth factors used, step procedure, duration and functional assay, if available.

To demonstrate the protocol section, we select a protocol differentiating human pluripotent cells (hES cells and iPS cells) into skeletal muscle-like progenitors [33], [34] (Figure 7) In this protocol, embryonic bodies (EBs) are formed and cells are induced to differentiate by conditional expression of *PAX7*. Upon induction, differentiated cells are sorted and proliferated to yield an expanded population of *PAX7* cells, which express myogenic progenitor markers such as *MYOG* and *MYH1*. These progenitors may be subsequently used to differentiate into myotubes *in vitro* or form mature skeletal muscle cells *in vivo*. The protocol card includes the title, description and available data, followed by an interactive clickable viewer describing each step of the protocol along with its source and target cells. Each of the protocol derived cells (PDCs) is clickable and will open the relevant information about this cell and the conditions used to derive it. The derivation steps are also shown on the viewer, and include the main growth factors used in each step, the main procedure performed (e.g. FACS) and the step duration. In the example protocol described, the hES cell line H9 was used as a source cell along with two other iPS cell clones (not shown). The resulting PDCs “Expanded *PAX7* cells” were matched to the *in vivo* myoblasts of the cervical-hypaxial-myotome [35].

### Regenerative Medicine

LifeMap Discovery provides information relating to regenerative medicine and cell-based therapy strategies as well. Cell-based therapy for neurodegenerative disease, heart disease, eye disease, type I diabetes, osteoarthritis etc., via replacement of damaged cells or tissues, and/or stimulation of the natural repair mechanisms of the body, offer great promise for near-future treatment of these conditions. Diseases associated with *in vivo* cells and anatomical compartments (e.g., congenital diseases) which are either related to development or represent potential targets for cell-based therapeutic approaches are also presented in LifeMap Discovery with links to the relevant entities, indicating whether a condition affects the presented entity or can potentially be treated by specific matched cultured cells. The full list of diseases related to *in vivo* entities is available at: http://discovery.lifemapsc.com/regenerative-medicine/diseases.

Cell-based therapeutic products are described at different stages of development (research, pre-clinical, clinical phases, and marketed). Each card describes a unique potential cell-based treatment approach for a specific disease or a group of related diseases. The information has been manually curated from various publications and/or selectively drawn from clinical trials resources. Each card is linked to the relevant cultured cells which were used as the source cells for the application. Since cultured cells are usually matched to *in vivo* cells or anatomical compartments, the cell therapy application also appears at the matched *in vivo* card and under its related organ, demonstrating the possibility to use specific cell- based treatment to cure diseases that affect the *in vivo* entities. Additional information, available in the cell-therapy application card includes the mode and regimen of cell delivery, mechanism of action, related animal models, pre-clinical and clinical information and related publications and links.

In addition, LifeMap Discovery provides specific links to MalaCards, the human disease database (http://www.malacards.org), enabling rapid access to information on relevant diseases such as description, clinical features, drugs and therapeutics, disease related gene expression, publications and more.

Available cell therapy applications, their related diseases and *in vitro* cells are available at: http://discovery.lifemapsc.com/regenerative-medicine/cell-therapy-applications.

### Data integration and data mining

In the previous sections, we described how LifeMap Discovery models developmental, stem cell biology and regenerative medicine applications, and the complex relations existing between the database elements. The database elements define the structure and intra-relations; each element is laden with either simple or complex data (e.g. development time, citations, microarray and signaling data). Data is usually cited or deep linked directly to specialized data resources, e.g. links from available genes expressed in a specific location/time will be linked to the corresponding *in situ* image. These different database elements are connected by computational and hand curated methods to enable both simple browsing and searching and more complex or data-driven associations.

The basic database functionality includes the ability to navigate the multi-dimensional space which is composed of cells linked to each other in a developmental and anatomy context, and the various classifications to anatomy, disease relations and *in vitro* analogs. LifeMap Discovery offers the following searching, browsing and graphical navigational options:

- Textual search – finding organs, tissues, cells and protocols, disease and cell therapy applications within the database.
- Gene search – finding where one or several genes are expressed.
- Detailed, sortable listing of all entities in all sections, and the ability to filter them as desired.
- Interactive graphical developmental navigator of *in vivo* cell and anatomical compartment development.
- Interactive graphical display of stem cell differentiation protocols.

We will focus on two useful features of data mining (i) gene search and (ii) matching of cultured stem and progenitor cells to *in vivo* cells and anatomical compartments that occur during mammalian development.

### Gene expression and Gene search

Gene expression data is most valuable for understanding function and classification of cells and tissues in the complex developmental stages and anatomical regions. LifeMap Discovery provides gene expression information at each of the three *in vivo* levels, cells, anatomical compartments and organ/tissues, as well as for *in vitro* cells (resulting from differentiation protocols). Gene search is available at http://discovery.lifemapsc.com/gene-expression-signals/gene-search.

**Figure 8:**
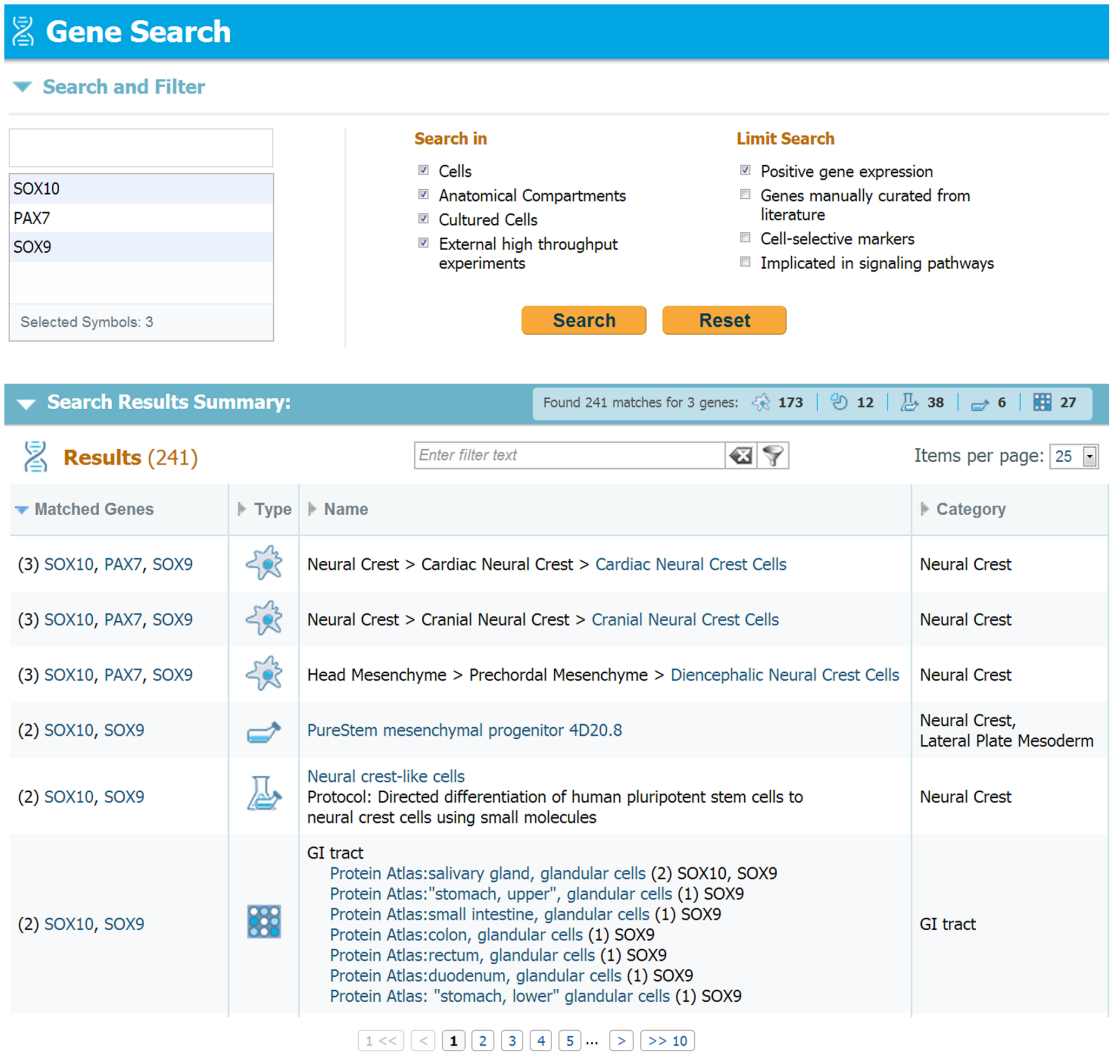
Gene search display. Demonstration of a gene search in LifeMap Discovery. Genes of interest are selected and the search can be limited and filtered by several criteria, e.g. the card type and the gene expression pattern. Following query submission, a results summary table is shown, indicating the number of results for each card type (cells, compartments and organ/tissue cards). The results list presents the name and number of genes found in each card, as well as the link to card itself.

Figure 8 shows the gene search panel, allowing for up to 10 genes to be searched simultaneously anywhere in the database. The genes of interest are typed and selected using gene symbols. Figure 8 demonstrates the search of 3 genes expressed in early migrating neural crest cells: *Sox10, Pax7* and *Sox9*.

The gene search can be limited by choosing the types of cards to be searched: cell cards, anatomical compartment cards, *in vitro* cultured cells and external resources (which include data on genes from high throughput gene expression datasets). The gene search can also be limited to specific types of genes or gene expression patterns: positively expressed, cell and lineage selective markers and genes that are implicated in signaling pathways. In the results summary section, results are provided in a table listing the occurrences of any of the genes on the search list, sorted by the number of genes found in the location and developmental time (e.g. 3/3 hits of neural crest cells). Columns are sortable and include the number of matched genes in each listed card, the card type: cell, anatomical compartment, *in vitro* cells and in external resources (e.g. [36],[37], [38]).

### Mapping *in vitro* cells to *in vivo* entities

The underlying assumption motivating the mapping effort is that stem or progenitor cells undergoing *in vitro* differentiation show similar gene expression patterns to their equivalent *in vivo* developmental state. Following that reasoning, knowledge of the exact or even approximate *in vivo* identity of a cell, i.e. it's correlation to the developmental ancestry tree and its anatomical location or tissue type classification can extremely useful. This type of information has been previously scattered in papers and specific databases, and difficult or impossible to associate or assemble with the developmental context.

While developing new protocols or following experimental procedures aimed at generating new classes of cells, such information empowers one to follow, plan, verify and target the *in vitro* differentiation process. When combined with the view and knowledge of all possible differentiation pathways, it becomes a roadmap to *in vitro* cell differentiation.

The matching process is calculated in five stages:

1. Database pre-processing: Available gene expression data that is stored in the LifeMap database in the various levels is analyzed (e.g. specific markers in cell cards, high throughput microarray data in cells or anatomical compartments etc.). In each database element, genes are scored based on overall or temporospatial abundance, available expression level or confidence. Genes are grouped or clustered based on similarity (unsupervised) or biological context (supervised). Each method and data type may yield a unique collection of sets of genes (geneset).
2. Sample preparation: The experimental gene expression data of the *in vitro* cell to be matched (7PEND24 in our example) is prepared. Raw data is normalized and a proper reference set is chosen. Depending on the data type (e.g. microarray vendor) the data is treated according to established analysis protocols to remove bad probes or treat multiple probes and samples. The various analysis and differential expression extraction methods are beyond the scope of this paper and described elsewhere (e.g. see the open access review and references within [39]), however the end result is a ranked list of genes expressed in that sample.
3. Matching: A number of methods and algorithms are applied to match the provided ranked gene list of step 2, to the various gene sets prepared in step 1. Each such process will yield a list of matches with scores and measures of statistical significance where available (e.g. P-values or FDR scores). One such method is using Gene Set Enrichment Analysis (GSEA) [40] to evaluate the likelihood of any constructed set of genes to show statistical significant presence in the tested phenotype (the ranked gene list).
4. Consolidation: The resulting geneset scores often show a wide selection of matches from the tested phenotype to other entities which are not trivial to interpret. Reasons include inherent, biological, temporal and measurement noise. For instance, the underlying gene expression data assimilated in LifeMap Discovery contains qualitative and quantitative data, obtained by numerous different technologies and different references, thus, the obtained results are not directly comparable. The computational process strives to reduce the noise and rank the matching results by aggregation and filtering. Each result has an associated set of scores for normalized match, significance and error measurements. In addition, each result can be broken down to the matched genes, their relative contribution in terms of expression, and their rank in the geneset. Results are thus aggregated based on tags and ordered by score with links to the genes and their scores.
5. Interpretation: Interpretation and assignment of matched cells is completed manually by reviewing the results and making sure they make biological sense.

## Conclusions

LifeMap Discovery has been created to bring essential and relevant knowledge from *in vivo* development into stem cell research and into clinical utility for future basic and applied research, including regenerative medicine applications. A deep understanding of the complex developmental paths, gene expression patterns in developing cells, and the signaling cascade that drives cellular differentiation, is invaluable for identification and classification of differentiated stem and progenitor cells, and for development of more accurate protocols for differentiating stem cells into desired target cells.

The unique value provided to the research community by LifeMap Discovery originates from:

- The ongoing *de novo* assembly, reconstruction, annotation and presentation of developmental paths and cell lineages, inferred from peer-reviewed literature.
- Manual assembly of gene expression and signaling molecules at each temporospatial cell and anatomical compartment.
- Collection and direct linking of high-throughput and large scale gene expression data.
- Computational analysis and matching of *in vitro* cells at various states of differentiations to similar *in vivo* cells, thereby identifying, classifying and indicating possible targets for research and clinical applications.
- Clinical relevance, associated diseases and differentiation protocols, providing a rich platform for therapeutic discovery.

LifeMap's long-term objective is the generation of the entire mammalian development atlas with cellular resolution. The goal is to bring to researchers high quality curated information on the cells, anatomical compartments and tissues/organs that comprise it, alongside *in vitro* and clinical data to accelerate stem and progenitor cell based therapeutics for the treatment of yet incurable conditions and diseases. The LifeMap Discovery platform offers information free of charge to academic institutions and will continue to grow and expand to related areas.
